# The Association of Depression and the Cardiovascular Risk Factors of Blood Pressure, HbA1c, and Body Mass Index among Patients with Diabetes: Results from the Translating Research into Action for Diabetes Study

**DOI:** 10.1155/2012/747460

**Published:** 2012-11-22

**Authors:** Lindsay B. Kimbro, W. Neil Steers, Carol M. Mangione, O. Kenrik Duru, Susan L. Ettner

**Affiliations:** ^1^Department of Medicine, David Geffen School of Medicine, University of California, 10940 Wilshire Boulevard, Suite 700, Los Angeles, CA 90025, USA; ^2^Department of Health Policy and Management, Jonathan and Karin Fielding School of Public Health, UCLA, Los Angeles, CA 90095, USA

## Abstract

Diabetic patients are nearly three times as likely to have depression as their nondiabetic counterparts. Patients with diabetes are already at risk for poor cardiovascular health. Using cross-sectional data from the translating research into action for diabetes (TRIAD) study, the authors tested the association of depression with cardiovascular risk factors in diabetic patients. Depression was measured using the patient health questionnaire (PHQ8). Patients who scored greater than 9 on the PHQ8 were classified as depressed and were compared with those who were not depressed (*n* = 2,341). Depressed patients did not have significantly different blood pressure levels than those who were not depressed. However, those who were depressed had higher HbA1c levels than those who were not depressed (*P* < 0.01) and higher BMIs than those who were not depressed (*P* < 0.01). These results indicate that depressed diabetic patients are at greater risk of having poor control of cardiovascular risk factors and suggest that depression screening should be a standard practice among this patient group.

## 1. Introduction

According to the Centers for Disease Control and Prevention, 9% of US adults meet the criteria for current depression while 3.4% meet the criteria for major depression [1]. As depression affects such a large portion of the US population, research investigating the risks it imposes is increasing. Depression has been linked to poorer control of risk factors for cardiovascular disease (CVD), including blood pressure, hemoglobin A1c, and body mass index [2–4]. CVD is one of the leading complications and underlying causes of death among persons with diabetes [5, 6]. Therefore, increased CVD risks associated with depression pose a particular threat to the diabetic population. Diabetics not only have poorer control of these risk factors as compared to nondiabetics, but are also nearly three times as likely to be depressed [7]. Depression poses a major risk to this vulnerable group. The question of whether the presence of depression is associated with increased blood pressure, HbA1c, and body mass index (BMI) among people with diabetes is largely unexamined, but studies that have analyzed these relationships are described later.

Of the nondiabetes specific studies that examined the relationship between blood pressure and depression, some found a decrease in blood pressure among those who are depressed [8, 9]. However, Nabi and colleagues showed that age-related risks of hypertension increase more within the depressed group than the nondepressed group, arguing that the risk of hypertension increases with multiple depressive episodes over time [10]. Few studies have examined whether depression is associated with HbA1c levels and those that did have mixed results. Some studies found an elevated HbA1c among diabetic patients with depression compared with their nondepressed counterparts [11]. One study of diabetic patients found that HbA1c is significantly correlated with depression for type 1 patients but not type 2 [12]. 

A few studies examined depression treatment and diabetic outcomes with mixed results, possibly due to the variation in side effects of antidepressant medications [4, 13–16]. Although the treatments positively affect mood, some could negatively affect CVD risk factors, thus negating the positive effects of mood improvement on these same factors. However, one study found that those who remained in depression remission 6 months following the completion of psychotherapy treatments showed significant improvements in HbA1c [14]. The relationship between depression and BMI among diabetics has been explored as well. Katon and colleagues showed that patients who were classified as having major or minor depression were significantly more likely to be obese than those without depression [17]. Another study, in a separate setting, yielded similar results [18].

 As depression affects a high percentage of persons with diabetes, it is important to understand whether the presence of depression, presumably due to undertreatment, is associated with poorer control of these intermediate cardiovascular risk factors. Using data from the translating research into action for diabetes (TRIAD) study, this report examines whether there is an association between depressive symptoms and cardiovascular risk factors among diabetics.

This study will be different from previous manuscripts in two ways. First, the TRIAD dataset is a large, geographically diverse dataset of diabetic patients. As the personal and medical health costs associated with poorer control over CVD risk factors are greater than their nondiabetic counterparts, it is important to analyze this group in particular for depression-related trends. Secondly, the patients within this cohort come from managed care settings. Therefore, the study provides an estimate of patient health information for those who are already connected to care but who may be lacking proper depression diagnoses and treatment. In the TRIAD dataset, only 44% of those who were identified as depressed by the PHQ-8 screener were taking antidepressant medications. We hypothesized that presence of depressive symptoms will be associated with poorer control of blood pressure, HbA1c, and body mass index.

## 2. Methods

### 2.1. Study Design and Participants

This study uses cross-sectional data for a diabetic sample to compare the outcomes of interest (blood pressure, HbA1c, and body mass index) among those with and without depression.

These analyses used data from the translating research into action for diabetes (TRIAD) study [19]. TRIAD is a multicenter prospective longitudinal study of persons with type 1 or type 2 diabetes in managed care settings. The study cohort consisted of enrollees from 10 health plans from 8 different states. Eligible patients were community dwelling, not pregnant, had diabetes for more than 1 year, spoke English or Spanish, were continuously enrolled in their health insurance plan for 18 months or more, used at least 1 diabetes-related medical service, and were able to provide informed consent. The institutional review boards at each participating site approved the study and all participants provided informed consent.

 This report specifically analyzes data from the 2003 wave (wave 2) of TRIAD in which depression was measured in the patient sample with diabetes. These analyses of wave 2 include data from five of the six centers contained in the original baseline survey. This sample includes those who were managed-care patients, 18 years of age and older, had diabetes, and were willing to participate in the wave 2 survey. Less than 5% of the sample had type 1 diabetes, defined as age <30 years and use of insulin but no oral hypoglycemic agents. The data were collected from patients using a mailed survey or computer-assisted telephone interviews and medical record reviews.

TRIAD originally enrolled a random sample of 11,927 adults, with 8,334 completing the initial survey and medical record review. While 6,760 patients completed the wave 2 survey, medical chart data were unavailable for 1,928 patients (28.5%), leaving 4,832 patients completing surveys with available medical charts. The sample size was further reduced by 1,279 patients due to missing data on at least one chart variable in our analyses, leaving 3,553 patients with complete chart data. The sample size was again reduced by 1,212 due to missing data on at least one survey variable in our analyses, leaving an analytic sample size of 2,341 with complete survey and chart data.

### 2.2. Variables

 There were three key outcome measures: Blood pressure, HbA1c, and body mass index (BMI). Blood pressure and HbA1c were obtained from the last recorded result of the patient medical record. BMI was generated using self-reported height and weight. These three variables were selected because of their relevance to cardiovascular health in diabetics as well as the completeness of the data within the TRIAD dataset. 

Depression was measured using the patient health questionnaire (PHQ-8) [20], in which anyone with a score of 10 or greater was classified as depressed and anyone with a score of 9 or less was classified as nondepressed. The Charlson comorbidity index was a covariate and was calculated from comorbidity data from the patients' medical records. Other variables used as covariates in the regression analyses included sex, age, race and ethnicity, income, education, insulin treatment, duration with diabetes, the Charlson Index, and whether the patient was married or living together with a partner, which came from the patient self-reported survey. All of the independent variables were categorical except for duration with diabetes and the Charlson Index and were represented using a series of indicators for each category, with one omitted reference category. Age was divided into 7 age categories that were 10 years apart. Income was divided into 4 categories that were $25,000 apart. These variables were categorized in order to discern any possible nonlinear trends and to ensure that there were enough participants in each category for the analysis. 

### 2.3. Statistical Analysis

Using linear regression to adjust for patient demographic and health variables as well as fixed research site effects, we compared the adjusted means between the depressed and nondepressed diabetic patients. Results are presented as adjusted means between the groups with and without depression with associated  *P*-values. SAS 9.2 was used to conduct the analyses. We examined sensitivity analyses looking at the associations of depression on the outcome variables after inclusion of smoking, physical activity, and antidepressant use.

## 3. Results

Descriptive statistics on outcomes and covariates in the depressed and nondepressed groups, and unadjusted tests of differences between depression groups on each of these variables, are shown in Table 1. HbA1c and BMI scores (but not blood pressure) were found to differ significantly between depression groups before regression adjustment. All covariates except duration with diabetes were found to differ significantly between the depressed and not depressed groups. It should be noted that 56% of those categorized as depressed had not taken antidepressant medication and might be considered undertreated in the absence of data on other treatments such as psychotherapy.

Differences between patients with and without depression in the three outcomes changed only minimally after adjusting for the above covariates.  Table 2 displays the results of the linear regressions, testing the adjusted associations of depression with the three outcomes. The regression coefficients in Table 2 show the amount of change in each outcome per single-unit change in each independent variable. For categorical independent variables, the coefficients indicate the value of the difference in the means of each outcome between the categories specified in the row of Table 2 and their reference category.

Depression did not show a significant association with blood pressure. Our results showed that blood pressure is more strongly associated with age and race than with any other independent variables. Most older age categories differed significantly from the reference category, while African Americans had higher systolic blood pressure levels than whites, and Latinos had lower systolic blood pressure levels than whites. This is consistent with previous study results [21, 22]. 

 Unlike blood pressure, HbA1c does have a significant adjusted association with depression. Diabetic patients with depression had HbA1c levels that were an average of 0.3 points higher than their nondepressed counterparts. Similar to blood pressure, age and race were also strongly associated with HbA1c, which decreased with age and was higher on average in minority groups. HbA1c also showed a significant trend downward with increasing education.

 Depression had a significant association with BMI, with depressed patients having an average BMI that was 1.55 units higher than that of nondepressed diabetic patients. Gender, age, race, and education also showed significant associations with BMI. 

 Sensitivity analyses were conducted including physical activity and smoking. The results showed only a small change in magnitude for blood pressure and no change for HbA1c. There was a moderate change in magnitude on the association of depression on BMI, but the sign and significance of depression did not change. As these results did not change our conclusions, we only show the original analyses. 

## 4. Discussion

 To summarize, among the diabetic patients in the TRIAD cohort, presence of depression is associated with poorer control of HbA1c and BMI. However, persons with depression did not, on average, have poorer blood pressure control than those without depression. In addition to adding evidence to the literature by analyzing a large and both geographically and ethnically diverse sample receiving care from multiple health plans and providers, our findings also show that despite having access to care, depressed diabetic patients still had a more difficult time managing these risk factors. 

Although the cross-sectional study design cannot establish causation with regard to the association of depression and control of cardiovascular risk factors, among persons with diabetes, those with depression may be less likely to maintain a healthy lifestyle of weight management and medication adherence [23]. In addition, these patterns of depression and poor control have been found to be cyclical. Luppino et al. found that depression and obesity are cyclically linked, and depression can lead to obesity while obesity can also lead to depression [24]. Also, Sacco and Bykowski [12] argue that a perceived lack of control over one's health, due to both poor adherence and adverse medical outcomes, can also be a cause of depression. This creates a cyclical pattern that may potentially be broken by appropriate screening, diagnosis, and treatment of depression. 

Our study has some limitations. First, our measure for depression was the PHQ-8 questionnaire, which is a screening test. Although the score is correlated with depression, it does not conclusively confirm the diagnosis. Second, our data do not allow for us to control patients who are receiving nonpharmacologic treatment for depression. Fewer than half of the depressed patients in our sample were treated with antidepressants, but others might have received other treatment modalities such as psychotherapy. Third, although the overall TRIAD study was longitudinal, this particular analysis was conducted using a single wave of data. Due to the cross-sectional nature of our analyses, we cannot ascertain whether depression causes a worsening of these cardiovascular risk factors over time, and the impact of previous and/or multiple episodes of depression cannot be captured. Fourth, we were unable to control other potentially important unmeasured factors such as family history of depression or other environmental factors. Finally, as our sample was limited to persons with diabetes with managed-care insurance, our findings may not generalize to the uninsured or those with other forms of coverage.

 The above results, along with other discussed complications from untreated depression [25], highlight the importance of screening all persons with diabetes for depression. Although others have shown that sustained improvement in depression from successful treatment can lead to an improvement in HbA1c and BMI, in the immediate term, treatment for depression is associated with an increase in HbA1c and blood pressure [8]. However, these results may be due to issues related to selection if patients with more severe depression and associated poorer CVD risk factor control are more likely to receive treatment for depression. Others have found positive results to depression treatment. Gois et al. found that sertraline and interpersonal psychotherapy both caused depression remission while also decreasing HbA1c levels [15]. Echeverry et al. also observed decreases in HbA1c and blood pressure when depressed diabetic patients were treated with sertraline [16]. These findings point toward the need for further research on the impact of various treatments of depression among persons with diabetes if we are to achieve the critically important goals of improving symptoms of depression and decreasing the risk of cardiovascular disease among this vulnerable group with multiple chronic conditions.

## Figures and Tables

**Table 1:** Distribution of variables associated with cardiovascular risk factors in study cohort.

Variable	*N*	Not depressed	Depressed*	*P* value
Total	2341	81.12%	18.88%	
Gender				<0.01
Female	1228	49.18%	66.52%	
Insulin treatment	473	19.06%	25.11%	<0.01
Married or living together	1457	64.77%	51.36%	<0.01
Age				<0.01
(18–34)	48	2.05%	2.04%	
(35–44)	192	7.58%	10.86%	
(45–54)	491	19.80%	26.02%	
(55–64)	669	28.44%	29.19%	
(65–74)	605	26.86%	21.49%	
(75–84)	317	14.48%	9.50%	
(85+)	19	0.79%	0.90%	
Race/ethnicity				<0.01
(White)	1402	60.61%	56.79%	
(Hispanic)	287	12.53%	11.09%	
(Black)	426	16.85%	23.98%	
(Asian/pacific islander)	102	4.84%	2.26%	
(Other)	124	5.16%	5.88%	
Income				<0.01
(<$15,000)	552	19.69%	40.27%	
($15,000–$40,000)	730	31.17%	31.22%	
($40,000–$75,000)	576	25.80%	19.46%	
(>$75,000)	483	23.33%	9.05%	
Education level:				<0.01
(< High school grad)	394	14.22%	28.05%	
(High school graduate)	640	26.38%	31.45%	
(Some college)	758	33.54%	27.38%	
(College grad or higher)	549	25.86%	13.12%	

Mean duration with diabetes (in years)	2341	12.81	13.44	0.22
Charlson index mean	2341	2.07	2.53%	<0.01

Unadjusted systolic blood pressure mean (mm Hg)	2341	135.6	135.7	0.94
Unadjusted A1C mean	2341	8.09	7.69	<0.01
Unadjusted body mass index mean	2341	34.33	31.35	<0.01

*Depressed is defined as a PHQ8 score of  >9.

**Table 2:** Linear regression estimates of the association of cardiovascular risk factors with depression.

Variable	Systolic blood pressure (mm Hg)		A1C		Body mass index	
	Coefficient estimate	*P* value	Coefficient estimate	*P* value	Coefficient estimate	*P* value
Depressed*	−0.33	0.75	**0.28**	**<0.01**	**1.55**	**<0.01**
Female	1.58	0.05	0.03	0.68	**2.26**	**<0.01**
Duration with diabetes	0.06	0.19	**0.01**	**<0.01**	−**0.04**	**0.03**
Insulin treatment	−0.11	0.92	**0.34**	**<0.01**	−**1.92**	**<0.01**
Married or living together	−0.23	0.80	0.01	0.94	−**1.01**	**0.01**
Charlson Index	−0.51	0.06	−0.03	0.27	0.11	0.28
Age (ref: 18–34)						
(35–44)	3.55	0.23	−**0.30**	**<0.01**	**4.35**	**<0.01**
(45–54)	**8.13**	**<0.01**	−0.29	0.25	**3.95**	**<0.02**
(55–64)	**11.35**	**<0.01**	−**0.69**	**<0.01**	**2.31**	**0.03**
(65–74)	**11.74**	**<0.01**	−**0.92**	**<0.01**	0.50	0.65
(75–84)	**13.08**	**<0.01**	−**1.15**	**<0.01**	−1.49	0.19
(85+)	**10.53**	**0.04**	−**1.38**	**<0.01**	−**6.56**	**<0.01**
Race/ethnicity (ref: white/caucasian)						
(Hispanic)	−**2.92**	**0.03**	**0.51**	**<0.01**	−0.21	0.68
(Black)	**5.38**	**<0.01**	**0.45**	**<0.01**	−**1.06**	**0.01**
(Asian/pacific islander)	2.37	0.22	**0.64**	**<0.01**	−**4.33**	**<0.01**
(Other)	1.06	0.54	0.28	0.06	−0.10	0.88
Income (ref: >$75,000)						
(<$15,000)	1.58	0.32	−0.19	0.18	−0.90	0.14
($15,000–$40,000)	1.36	0.28	−0.10	0.35	0.19	0.69
($40,000–$75,000)	0.77	0.51	0.16	0.12	0.03	0.95
Education level (ref: college grad or higher)						
(< High school grad)	0.43	0.76	**0.31**	**0.01**	0.99	0.07
(High school graduate)	1.70	0.14	**0.23**	**0.03**	**1.44**	**<0.01**
(Some college)	0.72	0.5	0.18	0.06	**1.23**	**0.003**

*Depressed is defined as a PHQ8 score of  >9. **Results in bold are significant at the 5% level.
